# Distinct roles of theta and alpha oscillations in the process of contingent attentional capture

**DOI:** 10.3389/fnhum.2023.1220562

**Published:** 2023-08-07

**Authors:** Chupeng Zhong, Yulong Ding, Zhe Qu

**Affiliations:** ^1^Department of Psychology, Sun Yat-sen University, Guangzhou, China; ^2^Key Laboratory of Brain, Cognition and Education Sciences (South China Normal University), Ministry of Education, Guangzhou, China; ^3^School of Psychology, South China Normal University, Guangzhou, China

**Keywords:** contingent attentional capture, neural oscillations, theta lateralization, alpha lateralization, N2pc

## Abstract

**Introduction:**

Visual spatial attention can be captured by a salient color singleton that is contingent on the target feature. A previous study reported that theta (4–7 Hz) and alpha (8–14 Hz) oscillations were related to contingent attentional capture, but the corresponding attentional mechanisms of these oscillations remain unclear.

**Methods:**

In this study, we analyzed the electroencephalogram data of our previous study to investigate the roles of capture-related theta and alpha oscillation activities. Different from the previous study that used color-changed placeholders as irrelevant cues, the present study adopted abrupt onsets of color singleton cues which tend to elicit phase-locked neural activities. In Experiment 1, participants completed a peripheral visual search task in which spatially uninformative color singleton cues were inside the spatial attentional window and a central rapid serial visual presentation (RSVP) task in which the same cues were outside the spatial attentional window. In Experiment 2, participants completed a color RSVP task and a size RSVP task in which the peripheral color singleton cues were contingent and not contingent on target feature, respectively.

**Results:**

In Experiment 1, spatially uninformative color singleton cues elicited lateralized theta activities when they were contingent on target feature, irrespective of whether they were inside or outside the spatial attentional window. In contrast, the same color singleton cues elicited alpha lateralization only when they were inside the spatial attentional window. In Experiment 2, we further found that theta lateralization vanished if the color singleton cues were not contingent on target feature.

**Discussion:**

These results suggest distinct roles of theta and alpha oscillations in the process of contingent attentional capture initiated by abrupt onsets of singleton cues. Theta activities may reflect global enhancement of target feature, while alpha activities may be related to attentional engagement to spatially relevant singleton cues. These lateralized neural oscillations, together with the distractor-elicited N2pc component, might consist of multiple stages of attentional processes during contingent attentional capture.

## 1. Introduction

Humans can voluntarily focus visual attention on a location or an object in the visual field to fulfill a visual task. Sometimes, however, our visual attention will be involuntarily captured by some irrelevant information, a phenomenon usually called attentional capture. Some studies claimed that a salient-but-irrelevant visual stimulus can capture attention in a pure bottom-up way (e.g., Theeuwes, 1992, 2004; Schreij et al., 2008). Alternatively, the hypothesis of contingent attentional capture argues that attentional capture is always mediated by top-down control and a stimulus attracts attention only when it possesses task-relevant features (Folk et al., 1992, 1994; Folk and Remington, 1998). For instance, when participants searched for a red target, a red singleton cue temporally preceding the target can capture attention [indexed by shorter reaction times (RTs) for targets presented at the cued locations], but the same singleton cue cannot capture attention when the target was defined by a smaller-sized stimulus (Eimer and Kiss, 2008).

To reveal the neural mechanisms of attentional capture, many researchers adopted electroencephalogram (EEG) recording technique and recorded the event-related potentials (ERPs) elicited by the task-irrelevant distractors. The most widely-used ERP index for attentional capture is the distractor-elicited N2pc (N2-posterior-contralateral) component. The N2pc is defined as an enhanced negativity over posterior electrodes contralateral to the side of a stimulus compared with the ipsilateral electrodes and is assumed to indicate attentional selection to that stimulus (Luck and Hillyard, 1994; Eimer, 1996; Kiss et al., 2008b; Luck, 2012). Evidence supporting bottom-up attentional capture mainly came from studies employing additional singleton paradigms developed by Theeuwes (1991, 1992). In a prior ERP study, Hickey et al. (2006) asked participants to search for a less salient shape singleton in a visual search array which sometimes contained a highly salient color singleton. They found that both the target singleton and the distractor singleton elicited N2pc components in trials when they appeared in opposite hemifields, but the distractor-elicited N2pc preceded the target-elicited N2pc. This distractor-elicited N2pc was recognized as a strong support for the hypothesis of bottom-up attentional capture (see also Burra and Kerzel, 2013; Barras and Kerzel, 2017; but see Jannati et al., 2013; McDonald et al., 2013 for results that salient distractors in additional singleton paradigms do not always elicit N2pc). In our recent N2pc studies, we found that after long-term perceptual learning, even a nonsalient shape could capture attention in a purely bottom-up manner (Qu et al., 2017; Hu et al., 2019). In contrast, evidence supporting contingent attentional capture usually adopted spatial cueing paradigms in which a cue array containing a spatially nonpredictive singleton always precedes a visual search array (Folk et al., 1992, 1994; Remington et al., 2001). Many studies showed that distracting cues with the search-guiding feature can capture attention (indexed by cue-elicited N2pc) but those without the search-guiding feature cannot (Eimer and Kiss, 2008, 2010; Kiss et al., 2008a; Leblanc et al., 2008; Ansorge et al., 2009; Brisson et al., 2009; Eimer et al., 2009; Lien et al., 2010; Kiss and Eimer, 2011; Wu et al., 2016; Goller et al., 2020).

Compared to the large number of N2pc studies, few studies focused on distractor-elicited neural oscillations in the process of attentional capture. Recently, an EEG study reported neural oscillation effects related to contingent attentional capture (Harris et al., 2017). In that study, participants were instructed to search for a letter T in a particular color among four colored T and then to identify its orientation. Preceding the target array, a cue array was presented which contained a spatially uninformative color singleton cue in target or non-target color. It was found that both the target-color and nontarget-color singleton cues elicited posterior theta (4–7 Hz) lateralization (with larger activities over contralateral brain region), and the magnitude of theta lateralization was greater in the matched compared to the non-matched cue condition. Besides, posterior alpha (8–14 Hz) lateralization (with smaller activities over contralateral brain region) was observed for target-color cue trials, but not for nontarget-color cue trials. Researchers inferred that the theta oscillations result from feature-based signal enhancement and the alpha lateralization reflects involuntary spatial attention shift. However, according to their designs and results, other explanations for the reported neural oscillations seem feasible as well.

The first concern is whether the theta oscillations are purely due to the attentional enhancement of task-relevant feature. Note that in the study of Harris et al. (2017), non-matched cues elicited theta lateralization to some extent as well, which cannot be perfectly explained by the account of feature-based enhancement. Also, the luminance of different types of color cues in their study was not controlled at a same level. Since both matched and non-matched cues were color singletons that had high physical saliency relative to other uniform cues, an alternative explanation cannot be excluded that cue-elicited theta oscillations in the study of Harris et al. (2017) might reflect or at least involve the encoding of unbalanced stimulus saliency in the cue array. The second concern is about the role of alpha lateralization in contingent attentional capture. The distractor-induced N2pc component, a classic neural index of attentional capture, is typically observed around 180–300 ms after stimulus onset (Luck and Hillyard, 1994; Eimer, 1996; Woodman and Luck, 2003; Luck, 2012). The cue-elicited alpha lateralization in the study of Harris et al. (2017), however, took effect at a period around 300–450 ms which seems relatively late to index the process of involuntary attention shift. Thus, it is unclear whether the observed alpha lateralization is related to early involuntary attention shift or later attentional processing (such as attentional engagement).

The current study aims to investigate the neural oscillations and to further clarify the roles of cue-elicited theta and alpha lateralization in the process of contingent attentional capture. We analyzed the data from two experiments initially reported in a prior ERP study (Huang et al., 2016). In these two experiments, participants completed two types of tasks. One was a peripheral visual search task, in which the cue arrays and target arrays were presented at the same peripheral locations. The task was to find a target with a specific color and to identify what the target is. The other was a central rapid serial visual presentation (RSVP) task, in which the cue arrays were presented at peripheral locations while the targets were located at the center. Participants were required to find a central target with a specific color or a larger size and to discriminate its identity. These two types of tasks adopted a same set of peripheral cue arrays among which color singleton cues and the other gray cues were controlled at a same luminance level. If the cue-elicited theta lateralization indeed reflects feature-based signal enhancement, one would expect to find this effect in both the peripheral visual search task and the central RSVP task when the targets shared the same color as the singleton cues, but not in the central RSVP task when the targets were defined by a different feature (i.e., larger size). As to the cue-elicited alpha lateralization, if it reflects involuntary spatial attention shift to peripheral singleton cues, we expect it to appear in both the peripheral search task and the central RSVP task when participants were searching for a same-color target, since we had found cue-elicited N2pcs that indexed involuntary attentional capture in both conditions (Huang et al., 2016). However, if it reflects later attentional engagement process, we expect to find little alpha lateralization in the central RSVP task, since under such a condition (i.e., a central target would never be presented at any peripheral cue location) later attentional engagement to a peripheral irrelevant singleton cue seems unnecessary.

## 2. Experiment 1

Using the spatial cueing paradigm, our previous study provided strong electrophysiological evidence (indexed by cue-elicited N2pc) that a peripheral color singleton cue captured attention when participants searched for a same-color target in either a peripheral search array or a central RSVP stream (Experiment 2 of Huang et al., 2016). By re-analyzing the data of this prior experiment, here we investigate whether there are neural oscillation activities (especially in theta and alpha bands) in the process of contingent attentional capture and whether these neural oscillation activities (if exist) would be modulated by task/attentional settings.

### 2.1. Materials and methods

#### 2.1.1. Participants

Twelve college students (mean age = 22 years, 8 females) participated in the experiment as paid volunteers. All but one of the participants were right-handed, and all of the participants had normal or corrected-to-normal vision. The sample size in our original study (Huang et al., 2016) was the same as that of the study of Eimer and Kiss (2008) which adopted a similar visual search task. Based on the statistics of Experiment 1 in the study Eimer and Kiss, we estimated the sample size needed to detect both a cue-elicited N2pc and a difference of N2pc between contingent and non-contingent cue conditions with the power of 0.80, and found the minimal sample size to be only 5.

#### 2.1.2. Stimuli and procedure

**FIGURE 1:**
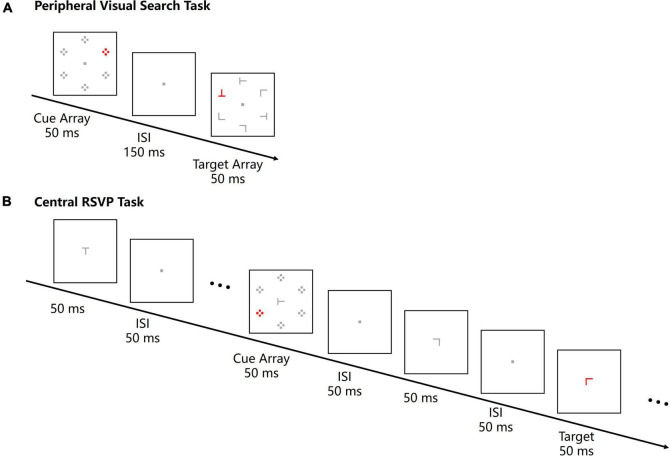
The stimulus sequences of a peripheral visual search task trial **(A)** and a central RSVP task trial **(B)** in Experiment 1. For both tasks, a cue array with a color singleton was presented for 50 ms and the cue-to-target SOA was 200 ms. The target was a red letter T or L, and participants were instructed to report the character of the target letter. All the stimuli are not drawn to scale.

Participants completed a peripheral visual search task and a central RSVP task at a distance of 100 cm to the screen in a dimly lit room. For the peripheral visual search task, a cue array was presented preceding the target array in each trial (Figure 1A). The cue array consisted of six sets of four dots. Each set of dots was 0.8° × 0.8° in size, and the center of each dot set was 4.3° away from a central fixation point. One set of dots was red and served as the color singleton cue, whereas the other sets were gray. The color singleton cue was presented at one of the four left or right locations randomly and equiprobably. The duration of the cue array was 50 ms. After a 150-ms interstimulus interval (ISI), the target array was presented at the same locations as the cue array, which meant that the cue-target stimulus onset asynchrony (SOA) was 200 ms. The target array consisted of five gray letters and one red target letter. Letters were T or L with varied rotation angles (0°, 90°, 180°, or 270°), and the size of each letter was 0.8° × 0.8°. The target letter was presented at one of the four lateral locations randomly and equiprobably as well. Since cue arrays and target arrays were presented at same peripheral locations and the task was to search for a peripheral target, the color singleton cues were inside the attentional window in the peripheral visual search task.

In the central RSVP task, an RSVP stream was presented at the center of the screen in each trial. The RSVP stream consisted of 12–15 letters (0.41° × 0.41°), each being presented for 50 ms with an ISI of 50 ms (Figure 1B). Letters were T or L with four possible rotation angles (0°, 90°, 180°, or 270°). Any temporally adjacent stimuli were different. All letters were gray except the red target letter. The target letter was randomly located at positions 8–11 in the stream, and there were always four letters following the target letter. A cue array, which was same as the one in the peripheral visual search task, was presented 200 ms before the target letter for 50 ms. Since the RSVP streams were always presented at the center of the screen and the task was to discriminate a central target, the peripheral singleton cues were outside the attentional window in the central RSVP task.

All the stimuli in these two tasks were equiluminant (11 cd/m^2^). In both tasks, participants were asked to discriminate the identity of the red target letter (T or L) by pressing the left or right response key. The order of tasks and mappings of target letters to response keys were counterbalanced across participants. Each task consisted of six successive blocks of 64 trials. Specifically, in the peripheral visual search task, there was 96 trials for each cue-target relation (same, vertically opposite, horizontally opposite, and diagonal); in the central RSVP task, there was 96 trials for each cue location (upper-left, upper-right, lower-left, and lower-right).

#### 2.1.3. Behavioral data analysis

In the peripheral visual search tasks, we divided trials into same-location condition and different-location condition (including vertically opposite, horizontally opposite, and diagonal conditions) according to the cue-target relations. The spatial cueing effect was computed by comparing the RTs and error rates in these two conditions. In the central RSVP task, we calculated the mean error rate of all trials.

#### 2.1.4. EEG recording and pre-processing

The EEG was recorded with an ANT EEG acquisition system (Refa-8 72-channel DC amplifier) from an array of 60 electrodes (58 scalp sites including FP1, FPz, FP2, AF3, AFz, AF4, F7, F3, Fz, F4, F8, FC5, FC3, FC1, FCZ, FC2, FC4, FC6, T7, C5, C3, C1, Cz, C2, C4, C6, T8, TP7, CP5, CP3, CP1, CPz, CP2, CP4, CP6, TP8, P7, P3, P5, P1, Pz, P2, P4, P6, P8, PO7, PO3, POz, PO4, PO8, O1, OZ, O2, I5, I3, Iz, I4, and I6 from 10-10 system, and the left and right mastoid). The horizontal and vertical electrooculograms (EOGs) were recorded as well. Horizontal EOGs were recorded by two electrodes positioned 1 cm away from the left and right outer canthi, and vertical EOGs were recorded by two electrodes placed above and below the left eye. The EEG was recorded with a common average reference online, and then was re-referenced to the average of the left and right mastoids offline. Electrode impedance was kept lower than 5 kΩ. The EEG and EOG signals were digitized at 512 Hz.

The offline EEG processing was performed using EEGLAB (Delorme and Makeig, 2004) and ERPLAB (Lopez-Calderon and Luck, 2014). For each participant, we first performed independent component analysis (ICA) on all scalp electrodes (except for the vertical and horizontal EOG sites) to remove components related to blinks and eye movements in continuous signals (that is, the HEOG and VEOG signals remained unchanged during this process). Epochs were then extracted from −500 to 1,200 ms relative to the onset of cue arrays, and signals from each epoch were corrected using a prestimulus baseline window from −200 to 0 ms. Any epoch exceeding a voltage of ±100 μV at any scalp electrode was rejected. Besides, to minimize the distortion of the poststimulus signal, epochs with blinks or eye movements that were close to the onset of cue array or target were rejected as well. We checked for blinks in the vertical EOG and rejected epochs with vertical EOG amplitudes exceeding ±70 μV. We checked for eye movements in the horizontal EOG by a split-half sliding window approach. A 400-ms time window was slid from −400 to 600 ms in steps of 10 ms. If the difference of mean voltages between the first and the second half of the sliding window exceeded 30 μV, the epoch was considered to contain an eye movement and then was rejected. On average, 17.3% (SD = 13.9%) of trials in the peripheral visual search task and 4.7% (SD = 6.1%) of trials in the central RSVP task were excluded from further analyses.

#### 2.1.5. Time-frequency analysis

To compute time-frequency representations, EEG signals from each artifact-free epoch were convolved with a family of complex Morlet wavelets with frequencies ranging from 0.7 to 68.5 Hz in 84 logarithmically spaced steps. The Morlet constant *m* (the number of cycles of each wavelet) was 4. The Morlet constant determines the frequency domain standard deviation *σ_*f*_* and time-domain standard deviation *σ_*t*_* of wavelets, which corresponds to the smoothing in the frequency domain and time domain, respectively. For each to-be-analyzed frequency *f*_0_, the frequency domain standard deviation (*σ_*f*_*) was equal to *f_0_/m*, while the time domain standard deviation (*σ_*t*_*) was 1/(2*πσ_*f*_*) (Tallon-Baudry et al., 1996). Thus, a larger Morlet constant *m* brings a larger temporal smoothing and a smaller frequency smoothing. Since we are mainly interested in the neural oscillations in lower frequency bands (theta and alpha) and their temporal information, we chose a relatively small *m* (i.e., 4) to ensure better temporal resolutions. The frequency bands of interest were the theta band (4–7 Hz) and the alpha band (8–14 Hz). In this study, the lower and upper bounds of the theta band were 4.05 and 6.68 Hz, while the alpha band ranged from 8.33 to 13.73 Hz. The frequency smoothing and the temporal smoothing of the central theta frequency (5.35 Hz) were 1.34 Hz and 0.119 s, respectively. For the central alpha frequency (11.00 Hz), the frequency smoothing and the temporal smoothing were 2.75 Hz and 0.058 s, respectively.

Convolution was done in the frequency domain. We first applied a fast Fourier transform to both wavelets and EEG signals, and then multiplied them in the frequency domain. Inverse fast Fourier transform was applied to the results of multiplication. The oscillation amplitude of each frequency at each time point was defined as the length of the complex vector resulting from the convolution. The amplitudes of oscillations in the theta band (4.05–6.68 Hz) were averaged to obtain the overall theta amplitude. Similarly, the overall alpha amplitude was obtained by collapsing amplitudes in the alpha band (8.33–13.73 Hz).

To further check whether the observed effects come from non-phase-locked (induced) activities, we obtained the amplitudes of non-phase-locked activities. To do so, separate ERPs were subtracted from each of the individual trials related to them (e.g., the left-cue ERP was subtracted from each left-cue trials), so that the phase-locked components were removed. Then we repeated the wavelet transform processing on these non-phase-locked signals.

#### 2.1.6. Lateralization index

To quantify the lateralization of oscillation activities time-locked to the onset of the color singleton cue, we calculated the lateralization index (LI) according to the following formula (Thut et al., 2006; Harris et al., 2017):


$$L\,I=\frac{A\,m\,p_{L\,e\,f\,t\,C\,u\,e}-A\,m\,p_{R\,i\,g\,h\,t\,C\,u\,e}}{A\,m\,p_{L\,e\,f\,t\,C\,u\,e}+A\,m\,p_{R\,i\,g\,h\,t\,C\,u\,e}}$$

In this formula, *Amp*_*LeftCue*_ and *Amp*_*RightCue*_ were the oscillation amplitude when color singleton cue was presented in the left and right visual fields, respectively. If the oscillation activities elicited by color singleton cue were stronger on the scalp electrodes contralateral to the location of the cue than those on ipsilateral electrodes, we would observe negative LI on the left hemisphere electrodes and positive LI on the right hemisphere. If the ipsilateral oscillation activities were stronger, LI would be positive on the left hemisphere electrodes and negative on the right hemisphere electrodes.

**FIGURE 2:**
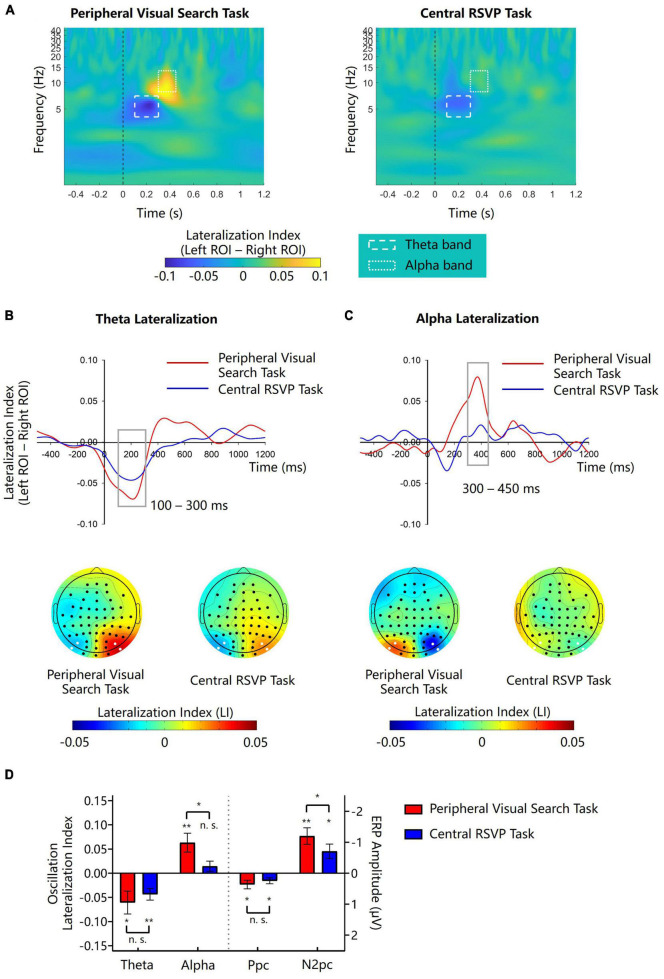
Lateralization of theta and alpha amplitudes in Experiment 1. **(A)** The left-minus-right lateralization index (LI) for the peripheral visual search task and the central RSVP task. Dash line boxes and dotted line boxes indicate theta and alpha bands and their time windows for analysis. **(B,C)** Waveforms and topographies of theta and alpha LIs for the peripheral visual search task and the central RSVP task. Gray boxes show the time window for analysis. White dots in topographies indicate ROIs. **(D)** The lateralization indexes of theta and alpha oscillations and the amplitudes of N2pc and Ppc in the peripheral visual search task and the central RSVP task. Error bars indicate standard errors. *p < 0.05, **p < 0.01, ^n.s.^not significant.

According to scalp topographies of theta and alpha LIs (Figures 2B, C), we respectively performed analyses on left and right regions of interest (ROIs) where theta lateralization and alpha lateralization were most evident. The left ROI included PO7 and I5 sites, and the right ROI included PO8 and I6 sites. We calculated the mean oscillation amplitudes of the two electrodes in the left and right ROIs respectively and then computed LIs for the two ROIs. The difference of LI between the two ROIs (left-minus-right) was considered as the measurement of lateralization magnitude. According to the waveforms of theta and alpha lateralization (Figures 2B, C), magnitudes of theta lateralization and alpha lateralization peaked at around 200 and 400 ms respectively, which was consistent to the study of Harris et al. (2017). We chose the mean theta LI in the 100–300 ms interval and the mean alpha LI in 300–450 ms for further analyses.

#### 2.1.7. Event-related potentials

Event-related potentials were obtained using the approach as reported in our previous study (Huang et al., 2016). Artifact-free EEG epochs were averaged for each cue location condition (left and right) and each task (peripheral visual search and central RSVP task), respectively. Then ERPs of scalp sites ipsilateral to the cue location were subtracted from ERPs of contralateral sites. To keep the N2pc results unchanged from our original study (Huang et al., 2016), we used the same electrodes (P7/P8 and PO7/8) for N2pc analyses where the cue-elicited N2pc was most evident. The contra-minus-ipsilateral ERPs at P7/P8 and PO7/PO8 were collapsed, and the mean amplitudes of N2pc were measured in the 180–230 time interval.

#### 2.1.8. Bayesian analyses

To examine whether some oscillation data provide evidence for null hypotheses in certain conditions, we conducted Bayesian analyses by JASP (version 0.17.1) with the default prior (*r* scale = 0.707). Following the widely accepted standard (Dienes and Mclatchie, 2018; van Doorn et al., 2021), we consider there is substantial evidence for null hypothesis (H0) over alternative hypothesis (H1) when Bayes factor (BF) is smaller than 0.33 (i.e., BF10 < 0.33).

### 2.2. Results

#### 2.2.1. Behavioral and ERP results

For the peripheral visual search task, the mean error rate was 4.67% (SD = 3.99%) for the same-location trials and 8.17% (SD = 6.92%) for the different-location trials. A paired samples *t*-test confirmed that the mean error rate was significantly lower in the same-location condition than in the different-location condition [*t*(11) = 2.473, *p* = 0.031, Cohen’s *d* = 0.714, 95% CI = [0.38%, 6.62%]). Consistently, the mean RT of the same-location trials (548 ± 51 ms, Mean ± SD) was significantly shorter than that of different-location trials (574 ± 58 ms; *t*(11) = 3.211, *p* = 0.008, Cohen’s *d* = 0.927, 95% CI = [8.01, 42.90]). These behavioral cueing effects indicated that spatial attention was successfully captured by peripheral singleton cues. For the central RSVP task, the mean error rate was 10.75% (SD = 4.54%).

**FIGURE 3:**
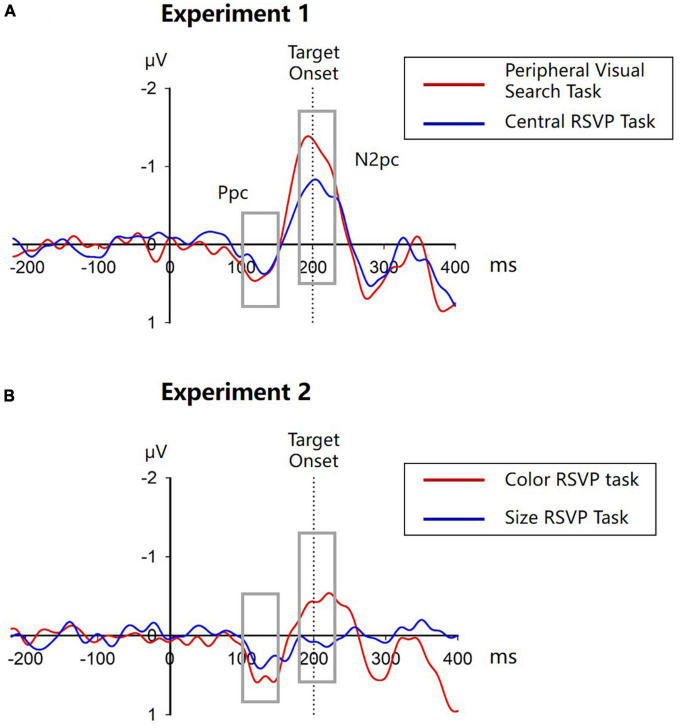
Contra-minus-ipsilateral ERPs in Experiment 1 **(A)** and Experiment 2 **(B)**. Vertical dotted lines indicate the onset time of targets. Gray boxes show the time window of Ppc (100–150 ms) and N2pc (180–230 ms).

Cue-elicited lateralized ERPs in both tasks are showed in Figure 3A. Note that in the peripheral visual search task, the target-elicited lateralized ERPs would be cancelled out when obtaining the cue-elicited lateralized ERPs because the target was randomly and equiprobably presented on the ipsilateral and contralateral side to the singleton cue, whereas in the central RSVP task, the central target could hardly elicit any lateralized ERPs. Thus, these lateralized ERPs would be solely elicited by the singleton cues. As reported in our previous study (Huang et al., 2016), singleton cues elicited obvious N2pcs in both the peripheral visual search task (−1.202 ± 0.918 μV, *t*(11) = −4.532, *p* = 0.001, Cohen’s *d* = −1.308, 95% CI = [−1.785, −0.618]) and the central RSVP task (−0.710 ± 0.810 μV, *t*(11) = −3.036, *p* = 0.011, Cohen’s *d* = −0.876, 95% CI = [−1.224, −0.195]), indicating that peripheral singleton cues captured attention irrespective of whether they were located inside or outside the spatial attentional window. Moreover, cue-elicited N2pc was significantly larger in the peripheral visual search task than in the central RSVP task (*t*(11) = −2.703, *p* = 0.021, Cohen’s *d* = −0.780, 95% CI = [−0.893, −0.091]; Figure 2D), indicating that attentional capture by peripheral singleton cues was stronger when these cues were inside compared to outside the attentional window.

#### 2.2.2. Theta lateralization

After the onset of the color singleton cue, there were obvious lateralized theta activities in both the peripheral visual search task and the central RSVP task (Figures 2A, B). The distributions (i.e., posterior area) and time windows (<300 ms) of the theta lateralization were similar in the two tasks (Figure 2B). Point-by-point one-sample *t*-tests showed that the theta lateralization was significant during 0–258 ms in the peripheral visual search task and during 4–297 ms in the central RSVP task (*p*s < 0.05). The theta LI was negative in the left ROI and was positive in the right ROI, suggesting that the theta activities were stronger on the posterior region contralateral to the location of the color singleton cue. Further analyses were based on the averaged LI in the 100–300 ms time window. One sample *t*-tests showed that the magnitudes of theta lateralization were significant in both task (peripheral visual search task: −0.061 ± 0.082, *t*(11) = −2.569, *p* = 0.026, Cohen’s *d* = −0.742, 95% CI = [−0.112, −0.009]; central RSVP task: −0.043 ± 0.042, *t*(11) = −3.587, *p* = 0.004, Cohen’s *d* = −1.035, 95% CI = [−0.069, −0.017]; Figure 2D). A paired samples *t*-test showed that, the magnitudes of theta lateralization in the two tasks did not significantly differ from each other (*t*(11) = −0.804, *p* = 0.438, Cohen’s *d* = −0.232, 95% CI = [−0.064, 0.030]; Figure 2D). Consistently, Bayesian analysis showed a nearly substantial evidence for no difference of theta lateralization between the two tasks (BF10 = 0.378).

As shown in Supplementary Figure 1A, there was no evident theta lateralization in the non-phase-locked activities. One-sample *t*-tests showed that theta lateralization of non-phase-locked activities was not significant in either the peripheral visual search task or the central RSVP task (*p*s > 0.492). Thus, the observed theta lateralization may mainly come from phase-locked theta activities.

#### 2.2.3. Alpha lateralization

In the peripheral visual search task, we observed obvious cue-elicited alpha lateralization in posterior sites (Figures 2A, C). Point-by-point one-sample *t*-tests revealed that alpha lateralization was significant during 306–436 ms in the peripheral visual search task (*p*s < 0.05). The alpha LI was positive in the left ROI and was negative in the right ROI (Figure 2C), suggesting that the amplitude of alpha oscillation was smaller on the contralateral sites than on the ipsilateral sites. But in the central RSVP task, no similar cue-elicited alpha lateralization was observed during the same period (Figures 2A, C). Further analyses were based on the averaged LI in the 300–450 ms time window. One sample *t*-tests showed that, the magnitude of alpha lateralization was significant during 300–450 ms in the peripheral visual search task (0.063 ± 0.068, *t*(11) = 3.245, *p* = 0.008, Cohen’s *d* = 0.936, 95% CI = [0.020, 0.106]), but not in the central RSVP task (0.014 ± 0.037, *t*(11) = 1.361, *p* = 0.201, Cohen’s *d* = 0.392, 95% CI = [−0.010, 0.038]; Figure 2D). Bayesian analysis showed that there was a bias toward no alpha lateralization in the central RSVP task (BF10 = 0.607). A further paired samples *t*-test showed that, the magnitude of alpha lateralization was significantly larger in the peripheral visual search task than in the central RSVP task (*t*(11) = 2.303, *p* = 0.042, Cohen’s *d* = 0.665, 95% CI = [0.002, 0.095]; Figure 2D).

Again, we did not observe evident alpha lateralization in non-phase-locked activities (Supplementary Figure 1B). One-sample *t*-tests showed that alpha lateralization of non-phase-locked activities was not significant in either the peripheral visual search task or the central RSVP task (*p*s > 0.128).

As shown in Figure 2A, no lateralization effect was evident in any other frequency band apart from the theta and alpha bands analyzed above.

#### 2.2.4. Further analyses

As shown in Figure 3A, there was an evident cue-elicited Ppc (posterior contralateral positivity) prior to the N2pc in the contra-minus-ipsilateral ERPs. Previous studies found that the singleton-elicited Ppc might be associated with salience computation (Fortier-Gauthier et al., 2012; Jannati et al., 2013; McDonald et al., 2023). To reveal the role of Ppc in the present study, we did further analyses. The mean amplitude of Ppc was measured in the 100–150 time interval using the same electrodes with N2pc. It turned out that the cue-elicited Ppc was significant in both the peripheral visual search task (0.364 ± 0.482 μV, *t*(11) = 2.613, *p* = 0.024, Cohen’s *d* = 0.754, 95% CI = [0.057, 0.670]) and the central RSVP task (0.243 ± 0.333 μV, *t*(11) = 2.529, *p* = 0.028, Cohen’s *d* = 0.730, 95% CI = [0.032, 0.454]). The amplitudes of Ppcs in these two tasks did not significantly differ from each other (*t*(11) = 0.931, *p* = 0.374, Cohen’s *d* = 0.269, 95% CI = [−0.165, 0.406]; Figure 2D). These results were consistent with previous findings, supporting that the cue-elicited Ppc might reflect the high salience of singleton cues, irrespective of whether they were inside the top-down attentional window or not. To examine whether there was a relation between the cue-elicited Ppc and theta lateralization, we performed exploratory tests for the correlation between them. Results showed that the correlation between the Ppc and theta lateralization was not significant in either the peripheral visual search task (*r*(11) = −0.059, *p* = 0.856) or the central RSVP task (*r*(11) = −0.439, *p* = 0.152).

Due to the facts that the theta and alpha activities were calculated from total power rather than induced/non-phase-locked activities, one might argue that these lateralized effects (especially the theta lateralization) might just reflect time-shifted N2pc components due to the wavelet transform. To test this hypothesis, we used notch-filtering methods to extract the theta (4–7 Hz) and alpha (8–14 Hz) activities from the ERPs respectively, and then examined whether the cue-N2pc still remained. Results showed that, after theta-band filtering, the cue-N2pc was still significant in either task (peripheral visual search task: −1.041 ± 0.930 μV, *t*(11) = −3.874, *p* = 0.003; central RSVP task: −0.571 ± 0.805 μV, *t*(11) = −2.454, *p* = 0.032; Supplementary Figure 5A). Although the cue-N2pc amplitudes decreased numerically after theta exclusion, the difference before and after theta exclusion did not reach a significant level under either task condition (both *p*s > 0.110). Results after alpha-band filtering also showed a significant cue-N2pc in either task (peripheral visual search task: −1.279 ± 1.059 μV, *t*(11) = −4.183, *p* = 0.002; central RSVP task: −0.789 ± 0.855 μV, *t*(11) = −3.195, *p* = 0.009; Supplementary Figure 6A). Again, the difference of cue-N2pc amplitudes before and after alpha exclusion did not reach a significant level under either task condition (both *p*s > 0.113). These results suggested that the cue-N2pc and cue-elicited theta (or alpha) lateralization did not originate from a same source of activities, but reflected different neural mechanisms (although their oscillation activities might have some extend of overlapping in certain frequency bands).

### 2.3. Discussion

In Experiment 1, peripheral color singleton cues elicited significant N2pc components when participants searched for a same-color target in either a peripheral visual search task or a central RSVP task, which was reported in our previous study (Huang et al., 2016). This is consistent with many previous ERP studies (e.g., Eimer and Kiss, 2008, 2010; Kiss et al., 2008a; Leblanc et al., 2008; Ansorge et al., 2009; Brisson et al., 2009; Eimer et al., 2009; Lien et al., 2010; Kiss and Eimer, 2011; Wu et al., 2016; Goller et al., 2020), indicating that distractors with the search-guiding feature can capture spatial attention involuntarily. Meanwhile, the color singleton cues elicited theta lateralization during early time windows in both tasks, with stronger amplitudes on contralateral sites. However, the magnitudes of theta lateralization showed no difference between the peripheral visual search task and the central RSVP task, indicating that it was not modulated by whether the cues were located inside or outside the attentional window. This is different from the pattern of N2pc results (Note that the cue-elicited N2pc was larger in the peripheral visual search task than in the central RSVP task). These results suggest that these two cue-elicited EEG indexes might reflect separate attentional processes in contingent attentional capture. Different from the widely accepted notion that cue-elicited N2pc indexes involuntary shift of attention to salient distractors (Hickey et al., 2006; Eimer and Kiss, 2008; Leblanc et al., 2008; Lien et al., 2008; Kiss et al., 2012; Luck, 2012), one speculation is that the theta lateralization might originate from global enhancement of attention to goal-related feature. The theta lateralization took effect in a short latency, also supporting this speculation. Similar speculation was also proposed by a prior study that only used a peripheral search task (Harris et al., 2017).

However, it is worth noting that color singleton cues were highly salient among cue arrays in both the peripheral visual search task and the central RSVP task. Thus, another plausible explanation of the singleton-cue-induced theta lateralization is that it might reflect the encoding of unbalanced physical saliency. That is, the observed theta lateralization was attributed to encoding an item with high physical salience, no matter whether its feature was contingent on the target or not. However, the results that there was no significant correlation between the theta lateralization and the salience-related Ppc in either task seem to go against this saliency-based hypothesis. Considering that the statistical power in correlation analyses might be insufficient, further study is needed to test this saliency-based hypothesis, especially under a circumstance that a color singleton cue is designed to be not contingent on the target feature.

In Experiment 1, peripheral color singleton cues elicited lateralized alpha oscillations (with smaller amplitudes over contralateral sites) in the peripheral visual search task but not in the central RSVP task. The result of alpha lateralization in the peripheral visual search task was consistent with a previous study (Harris et al., 2017) which adopted a similar peripheral task. Alpha lateralization (with smaller amplitudes over contralateral sites) was widely considered a neural marker of lateralized spatial attention allocation (Worden et al., 2000; Kelly et al., 2006; Thut et al., 2006; Rihs et al., 2009; Noonan et al., 2016; Foster et al., 2017). Now that the peripheral color singleton cues could capture attention (indexed by the cue-elicited N2pc) in the central RSVP task, why were the same singleton cues not able to elicit alpha lateralization? Our results suggested that the inside/outside relation to attentional window is a crucial factor for the appearance of cue-elicited alpha lateralization. Different from the peripheral visual search task where the singletons cues were presented inside the attentional window, the same cues were kept to be outside the attentional window in the central RSVP task. We hypothesize that alpha lateralization might not reflect the process of the attentional shift itself (reflected by cue-elicited N2pc), but the process of attentional engagement afterward. This hypothesis was also supported by the fact that alpha lateralization took effect at a later period (300–450 ms) relative to cue-elicited N2pc (180–230 ms). In the central RSVP task, although the peripheral singleton cues could capture attention to some extent (indexed by a moderate cue-elicited N2pc), there might be little attentional engagement process to these distractors which were outside of attentional window, resulting in little alpha lateralization.

## 3. Experiment 2

In Experiment 1, theta lateralization was elicited by color singleton cues in tasks requiring identification of a same-color target. However, theta lateralization may reflect encoding of unmatched physical saliency rather than attentional enhancement of the target color. To exclude this explanation, in Experiment 2, we further investigated whether a same pattern of theta lateralization could be still observed when the same singleton cues were not contingent on the target feature. In addition, no significant alpha lateralization was elicited by peripheral singleton cues in the central RSVP task with a relatively small effect size. Thus, another issue we tried to confirm in Experiment 2 is that the peripheral cues indeed cannot elicit alpha lateralization during 300–450 ms in a central RSVP task. Here, we chose to re-analyze the data from Experiment 3 of Huang et al. (2016). In this experiment, participants completed two types of central RSVP tasks. The same set of color singleton cues was contingent on the target feature in a central color RSVP task, but not in a central size RSVP task.

### 3.1. Materials and methods

#### 3.1.1. Participants

Twelve college students (mean age = 21 years, 5 females) participated in the experiment as paid volunteers. All but one of the participants were right-handed, and all of the participants had normal or corrected-to-normal vision.

#### 3.1.2. Stimuli and procedure

**FIGURE 4:**
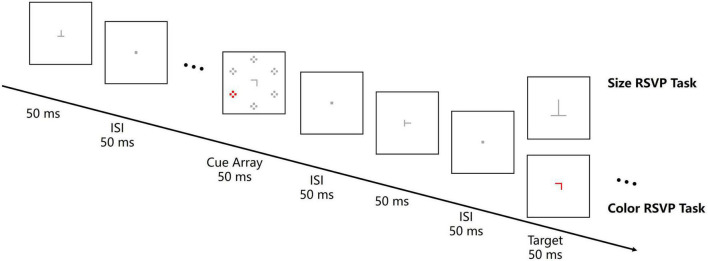
Illustration of a central RSVP tasks trial in Experiment 2. The central RSVP stream consisted of 12–15 letters (rotated T or L). Letters were presented for 50 ms with an ISI of 50 ms. A cue array with a color singleton was presented for 50 ms and the cue-target SOA was 200 ms. Participants were instructed to report the character of the red (color RSVP task) or the larger-sized (size RSVP task) target letter. All the stimuli are not drawn to scale.

Participants completed two central RSVP tasks at a distance of 100 cm to the screen in a dimly lit room. One of the tasks was a central color RSVP task, which was the same as that in Experiment 1 except that the size of central letters varied from 0.18° × 0.18° to 0.45° × 0.45°. The other task was a central size RSVP task (Figure 4). In this task, participants were instructed to discriminate the identity of a larger-sized target letter (T or L) by pressing left or right response key. The distractor letters in the RSVP stream was 0.36° × 0.36°, and the larger-sized target letters varied from 0.52° × 0.52° to 0.56° × 0.56° to keep participants’ error rate comparable as in the central color RSVP task. The color singleton cue was not contingent on the target feature in the central size RSVP task since the target was defined as a larger size rather than a same color. Each task consisted of six successive block of 64 trials. Thus, there was 96 trials for each cue location (upper-left, upper-right, lower-left, and lower-right). The order of tasks and mappings of target letters to response keys were counterbalanced across participants.

#### 3.1.3. EEG recording, pre-processing, ERP, and time-frequency analysis

The EEG recording and preprocessing were identical to those in Experiment 1. On average, 9.8% (SD = 13.3%) of trials in the central color RSVP task and 5.0% (SD = 6.7%) of trials in the central size RSVP task were excluded from further analyses. Then the same wavelet transform as in Experiment 1 was applied to each artifact-free trial. Cue-elicited ERPs were calculated in the same way as in Experiment 1.

### 3.2. Results

#### 3.2.1. Behavioral and ERP results

Mean error rates were 9.58% (SD = 2.76%) and 9.20% (SD = 2.29%) for the central color RSVP task and the central size RSVP task, respectively. A paired samples *t*-test showed no significant difference of error rates between the two tasks (*t*(11) = 0.557, *p* = 0.589, Cohen’s *d* = 0.161, 95% CI = [−1.07%, 1.80%]).

**FIGURE 5:**
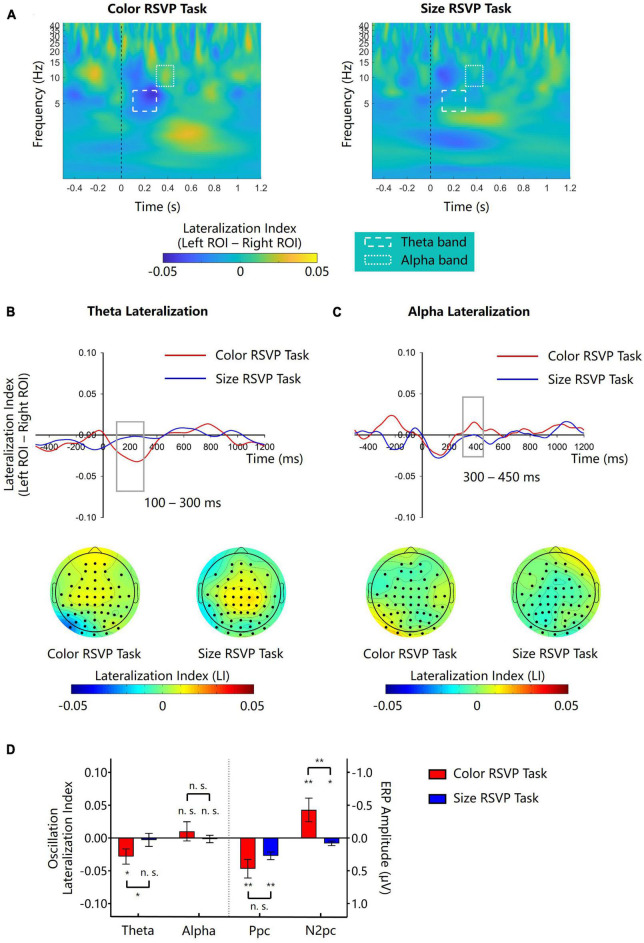
Lateralization of theta and alpha amplitude for Experiment 2. **(A)** The left-minus-right lateralization index (LI) for the color and the size RSVP task. Dash line boxes and dotted line boxes indicate theta and alpha bands and their time windows for analysis. **(B,C)** Waveforms and topographies of theta and LI for the color and the size RSVP task. Gray boxes show the time window for analysis. White dots in topographies indicate ROIs. **(D)** The lateralization indexes of theta and alpha oscillations and the amplitudes of N2pc and Ppc in the color and size RSVP tasks. Error bars indicate standard errors. *p < 0.05, **p < 0.01, ^n.s^.not significant.

Cue-elicited ERPs are showed in Figure 3B. A cue-elicited N2pc can be observed in the central color RSVP task, but not in the central size RSVP task. A one-sample *t*-test confirmed that the N2pc was significant in the central color RSVP task (−0.429 ± 0.622 μV, *t*(11) = −2.388, *p* = 0.036, Cohen’s *d* = −0.689, 95% CI = [−0.824, −0.034]). But in the central size RSVP task, there was a small Pd (distractor positivity) in the 180–230 ms interval (0.082 ± 0.114 μV, *t*(11) = 2.467, *p* = 0.031, Cohen’s *d* = 0.712, 95% CI = [0.009, 0.154]), which might reflect slight suppression to the peripheral color singleton cues when they are task irrelevant. The mean amplitudes during the N2pc time interval were significantly different between the two tasks (*t*(11) = 2.752, *p* = 0.019, Cohen’s *d* = 0.794, 95% CI = [−0.918, −0.102]; Figure 5D).

#### 3.2.2. Theta lateralization

In the central color RSVP task, we found similar cue-elicited theta lateralization as in Experiment 1 (Figure 5A, left). The scalp topography of theta LI suggested that theta activities was stronger on the posterior contralateral region (Figure 5B). Point-by-point one-sample *t*-tests showed that theta lateralization was significant during 174–328 ms (*p*s < 0.05). A further one-sample *t*-test showed that the theta lateralization was significant in 100–300 ms (−0.028 ± 0.040, *t*(11) = −2.432, *p* = 0.033, Cohen’s *d* = −0.702, 95% CI = [−0.054, −0.003]; Figure 5D). However, in the central size RSVP task, the same color singleton cues did not elicit significant theta lateralization during the same time window (−0.004 ± 0.034, *t*(11) = −0.284, *p* = 0.782, Cohen’s *d* = −0.082, 95% CI = [−0.025, 0.019], BF10 = 0.298; Figure 5A, right). A paired samples *t*-test showed that the difference of theta lateralization during 100–300 ms between the two tasks was significant (*t*(11) = −2.697, *p* = 0.021, Cohen’s *d* = −0.779, 95% CI = [−0.046, −0.005]; Figure 5D).

As in Experiment 1, the theta lateralization in the color RSVP task was not significant in the non-phase-locked activities (*t*(11) = −0.849, *p* = 0.414, Supplementary Figure 2A).

#### 3.2.3. Alpha lateralization

During 300–450 ms after the onset of color singleton cue, no obvious alpha lateralization was observed in either the central color RSVP task or the central size RSVP task (Figures 5A, C). One-sample *t*-tests on the mean magnitude of alpha lateralization in the 300–450 ms time window confirmed that the alpha lateralization was not significant in either of these two tasks (color task: 0.010 ± 0.051, *t*(11) = 0.702, *p* = 0.782, Cohen’s *d* = 0.203, 95% CI = [−0.022, 0.042], BF10 = 0.355; size task: −0.001 ± 0.020, *t*(11) = −0.252, *p* = 0.805, Cohen’s *d* = −0.073, 95% CI = [−0.014, 0.011], BF = 0.295; Figure 5D). Since the color central RSVP task was almost identical to that in Experiment 1, we merged the data of the two experiments and confirmed a nonsignificant alpha lateralization effect in the color RSVP task (0.012 ± 0.043, *t*(23) = 1.398, *p* = 0.175, Cohen’s *d* = 0.285, 95% CI = [−0.006, 0.031]; BF10 = 0.387).

In other frequency bands, we found no evident lateralization effect either.

#### 3.2.4. Further analyses

As in Experiment 1, the cue-elicited Ppc was significant in both the color RSVP task (0.468 ± 0.492 μV, *t*(11) = 3.297, *p* = 0.007, Cohen’s *d* = 0.952, 95% CI = [0.156, 0.780]) and the size RSVP task (0.271 ± 0.203 μV, *t*(11) = 4.624, *p* = 0.001, Cohen’s *d* = 1.335, 95% CI = [0.142, 0.399]), with no significant difference between each other (*t*(11) = 1.187, *p* = 0.260, Cohen’s *d* = 0.343, 95% CI = [−0.168, 0.563]; Figure 5D). These results further supported that the Ppc reflected the salience computation of the singleton cues, irrespective of whether they had the search-guiding feature or not. Again, the correlation between the Ppc and theta lateralization was not significant in the color RSVP task (*r*(11) = −0.095, *p* = 0.769), supporting that they might result from different underlying mechanisms.

Like in Experiment 1, we extracted the theta (4–7 Hz) and alpha (8–14 Hz) activities from the ERPs respectively through notch-filtering methods, and then examined whether the cue-N2pc still remained in the color task. Results showed that, after theta-band filtering, the cue-N2pc was still significant in the color task (−0.437 ± 0.644 μV, *t*(11) = −2.352, *p* = 0.038; Supplementary Figure 5B), and the difference of cue-N2pc before and after theta exclusion did not reach a significant level (*p* = 0.133). In the size task, however, there was no significant cue-N2pc (0.042 ± 0.345 μV, *t*(11) = 0.417, *p* = 0.685). Results after alpha-band filtering also showed a significant cue-N2pc in the color task (−0.632 ± 0.758 μV, *t*(11) = −2.889, *p* = 0.015; Supplementary Figure 6B) but not in the size task (−0.046 ± 0.315 μV, *t*(11) = −0.507, *p* = 0.622). Again, the difference of cue-N2pc amplitudes before and after alpha exclusion was not significant (*p* = 0.686). These results confirmed that the cue-N2pc and cue-elicited theta lateralization did not originate from a common source of activities.

### 3.3. Discussion

In the central color RSVP task of Experiment 2, color singleton cues elicited significant theta lateralization in the 100–300 ms time window, which replicated the findings of Experiment 1. More importantly, such cue-elicited theta lateralization was only observed in the central color RSVP task but not in the central size RSVP task. Note that the cue arrays were identical in both tasks. Thus, our results indicated that the cue-elicited theta lateralization was due to the singleton cue’s contingency on the target feature rather than its high physical saliency. The nonsignificant correlation between the theta lateralization and salience-related Ppc also supported this inference. Similar to Experiment 1, no alpha lateralization was observed in the 300–450 ms time window for a peripheral feature-matched cue, reinforcing a crucial role of its inside/outside relation to spatial attentional window on cue-elicited alpha lateralization. These results supported the notion that the underlying mechanisms of cue-elicited theta and alpha lateralization may be different.

## 4. General discussion

The current study investigated the mechanisms of theta and alpha neural oscillation activities in the process of contingent attentional capture. Contingent singleton cues elicited higher theta activities on the posterior region of the hemisphere contralateral to their spatial positions when the cues were preceding the targets, irrespective of whether the cues were inside or outside the attentional window. Such theta lateralization disappeared when the feature of the singleton mismatched the target feature. In addition, contingent singleton cues can also elicit lateralized alpha activities, but only when they were presented inside the attentional window. These results suggest different roles of theta and alpha oscillations in the process of contingent attentional capture.

The cue-elicited theta lateralization may reflect global enhancement of goal-related feature. According to the contingent attentional capture theory, when humans are searching for a specific target, an attentional control setting will be formed and provide an enhancement to the goal-related feature (Folk et al., 1992; Folk and Remington, 1998). This goal-driven enhancement of the attentional control setting is selective, therefore those non-matched features will not be enhanced. In line with the contingent attentional capture theory, the non-contingent singletons did not elicit any theta lateralization in the current study. Besides, it is also claimed that goal-driven feature enhancement occurs quite early, even before the initial spatial attention shift (Folk et al., 1992; Folk and Remington, 1998). In the present study, the theta lateralization took effect soon after the onset of singleton cues, which is consistent with this claim. Most visual search models, including the priority map model (Fecteau and Munoz, 2006) and the Guided Search model (Wolfe, 1994, 2021), proposed that simple features in the whole visual field will be encoded in the pre-attention stage. The attentional control setting may globally enhance all the stimuli with the goal-related feature. This can be supported by numerous studies that goal-directed feature-based attention will enhance all the stimuli with the attended feature in the entire visual field (e.g., Saenz et al., 2002; Sàenz et al., 2003; Zhang and Luck, 2009; White and Carrasco, 2011; Painter et al., 2014; Bartsch et al., 2018; for reviews, see Maunsell and Treue, 2006; Liu, 2019). It is thus reasonable that cue-elicited theta lateralization could be observed in both the peripheral visual search task and the central RSVP task, because the goal-related feature will be enhanced irrespective of whether it is presented inside or outside the attentional window. The feature enhancement speculation of posterior theta lateralization is compatible with previous findings that large posterior theta lateralization can be elicited by informative spatial cues (Green and McDonald, 2008), uninformative contingent spatial cues (Harris et al., 2017), and targets of visual search (Dowdall et al., 2012).

In the present study, feature-matched singleton cues elicited obvious alpha lateralization when they were inside the attentional window (i.e., in the peripheral search task), but such alpha lateralization disappeared when the same cues were outside the attentional window (i.e., in the central RSVP task). Traditionally, alpha lateralization was considered to reflect voluntary attentional allocation to an item in the left or right side of the visual field (Thut et al., 2006; Noonan et al., 2016; Foster et al., 2017; Foster and Awh, 2019). Recently, a study linked alpha lateralization to involuntary capture of spatial attention (Harris et al., 2017). However, this involuntary capture inference of alpha oscillation cannot explain the disappearance of alpha lateralization in the present color RSVP task, since the cue-elicited N2pc indicated that attention had been successfully captured by those peripheral singleton cues (Huang et al., 2016). Besides, the alpha lateralization (300–450 ms) in the present study emerged later than the N2pc (180–230 ms), suggesting that the alpha lateralization may be related to some slow attentional process following the attention shift. Another well-known theory about alpha lateralization is that alpha lateralization results from increased suppression to to-be-ignored locations (Worden et al., 2000; Kelly et al., 2006; Rihs et al., 2009; Jensen and Mazaheri, 2010; Foxe and Snyder, 2011; Payne and Sekuler, 2014). The alpha suppression can be revealed by differences between pre- and post-stimulus alpha amplitudes contralateral to the to-be-ignored location (a larger alpha amplitude in post-stimulus compared to pre-stimulus interval means the existence of alpha suppression; Kelly et al., 2006; Rihs et al., 2009; Gundlach et al., 2020), by differences of alpha activities between different cue/distracting conditions (alpha suppression exists if alpha amplitude is larger when a cue/distractor is presented laterally than when a cue/distractor is presented vertically or not presented at all; Green and McDonald, 2010; Forschack et al., 2022), or by differences of increment between alpha activities ipsilateral and contralateral to the to-be-ignored location (faster increasing of alpha activities at contralateral locations indicates alpha suppression; Worden et al., 2000; Green and McDonald, 2010). In the peripheral visual search task of Experiment 1, we found that both alpha amplitudes contralateral and ipsilateral to singleton cues were lower than the pre-stimulus baseline and were still decreasing in the time interval of lateralization analysis (Supplementary Figure 3B). Therefore, it seemed insufficient to attribute the alpha lateralization observed in present study to suppression to the locations contralateral to singleton cues.

We propose that the alpha lateralization in the current study may reflect spatial attention engagement which followed attentional shift. Spatial attention engagement refers to the spatial enhancement of visual stimuli at the attended location for further processing (Nieuwenstein et al., 2005; Folk et al., 2009; Zivony and Lamy, 2018). This speculation does not contradict to the traditional alpha lateralization studies (Worden et al., 2000; Sauseng et al., 2005; Kelly et al., 2006; Thut et al., 2006; Rihs et al., 2009; Jensen et al., 2012; Klimesch, 2012), since in those studies spatial attention was usually voluntarily allocated to a specific location/object, and very likely involved attentional engagement (and further identification) process. For instance, a recent study showed that the crowding level of target arrays can modulate the target-elicited alpha lateralization (Bacigalupo and Luck, 2019), suggesting that alpha oscillations may be associated with stimulus identification. On the other hand, several studies found that attentional capture is not necessarily followed by attentional engagement (Zivony and Lamy, 2018; Maxwell et al., 2021). For instance, in the study of Zivony and Lamy (2018), although both relevant-color and irrelevant-color onset cues can capture attention, the response-compatibility effect can be only observed in relevant-color cue trials, indicating little attentional engagement to those irrelevant-color cues. Similarly, in the present central color RSVP task, although peripheral singletons cues captured attention to some extent (indexed by a moderate cue-elicited N2pc), there would be unnecessary to initiate following attentional engagement to those cues because the central targets would never appear in those peripheral locations, which resulted in the disappearance of cue-elicited alpha lateralization. Different from our hypothesis that cue-N2pc reflects attentional shift and alpha lateralization reflects attentional engagement, a recent study (Zivony et al., 2018) claimed that cue-elicited N2pc reflected attentional engagement rather than attentional shift. We speculate that the interpretational discrepancy might mainly result from different experimental designs in the two studies. The study of Zivony et al. (2018) adopted an attentional blink paradigm in which participants were required to fulfill both a T1 (first target, presented in both streams) task and T2 (second target, presented in one of the streams) task. Between the T1 and T2, there was a cue presented on either the left or the right stream. Such a two-task design would lead to allocation of top-down attention to both streams throughout the task. Since participants already allocated their spatial attention to the exact cue locations before the cues, it would be reasonable that little attentional shift was needed from the center to the cue location, leading to that the cue-N2pc mainly reflects “onset of attentional engagement” (as stated in their study) rather than transient attentional shift. By contrast, in our study, the location of a singleton cue is randomly selected from multiple location candidates and is not known to participants beforehand (see also Eimer and Kiss, 2008; Leblanc et al., 2008; Ansorge et al., 2009; Wu et al., 2016; Goller et al., 2020). Thus, the peripheral feature-matched cues would tend to cause attentional shift from the center to the cue location (indexed by cue-N2pc) and possible attentional engagement (indexed by alpha lateralization).

Based on the inferences above, we propose that there are multiple attentional stages in the process of contingent attentional capture. First, all the stimuli in the visual field are encoded in a feature-based manner. Top-down attentional settings enhance the goal-relevant feature globally (Folk et al., 1992; Folk and Remington, 1998), making all potential items with relevant features higher priority to be selected (Wolfe, 1994, 2021; Fecteau and Munoz, 2006). In our contingent attentional capture paradigms, feature-matched distractors always precede targets, so these matched distractors will be enhanced first. The cue-elicited theta lateralization with a short latency will occur in this stage. Second, attention selects the stimulus that is most likely to be the target. Actually, in the contingent attentional capture circumstance, the first selected stimulus would be the feature-matched distractor rather than the target, which is indexed by the cue-elicited N2pc. The modulation of top-down spatial attention on attentional capture may take effect in this stage. If a feature-matched distractor is presented at a location known *a priori* as impossible to contain a target, it will be harder to shift attention focus to that location. Such a difficulty of attentional shift may lead to a smaller distractor-elicited N2pc component (Huang et al., 2016; Su et al., 2020). Third, spatial attention engages in the location of the selected stimulus for further visual processing, which will produce alpha lateralization in a later period. It is not an obligatory stage in attentional capture because attention focus can withdraw from the selected stimulus immediately if it is highly unlikely to be a target (Boot and Brockmole, 2010; Blakely et al., 2012). Only if peripheral singleton cues appear at spatially relevant locations where targets are also probably presented [such as in peripheral visual search task in the present study and the study of Harris et al. (2017)], spatial attention might be engaged to these cues to some extent, resulting in cue-elicited alpha lateralization. The occurrence of attentional engagement may be one of the sources of behavioral capture effects, which is in line with the finding that contingent attentional capture effects were larger in the identification task than in the detection task (Schönhammer and Kerzel, 2017).

In the present study, the cue-elicited theta and alpha lateralization mainly came from phase-locked oscillatory activities, whereas in the study of Harris et al. (2017) the reported theta and alpha lateralization were from induced/non-phase-locked activities. We speculate that the discrepancies of results between studies might mainly be due to the difference in experimental designs, especially the methods of cue presentation. In our study, the cue arrays are abrupt onsets which last for a very short period of time (50 ms). This transient onset cue may generate a strong phase-resetting effect on neural oscillations over a relatively wide frequency range (including theta and alpha bands), which is similar to the effects in many behavioral oscillation studies that a transient onset cue can reset the phase of behavioral oscillations (e.g., Landau and Fries, 2012; Fiebelkorn et al., 2013; Song et al., 2014). This phase-resetting effect of abrupt cues would cause most of the oscillatory activities in a phase-locked manner. In the study of Harris et al. (2017), however, the cues are color-changed placeholders. This type of cues may have less intensities compared to abrupt onsets, probably leading to a weaker phase-resetting effect and more non-phase-locked oscillatory activities. Further studies are needed to confirm the impact of cue presentation on the mode of phase-resetting in neural oscillations.

## 5. Conclusion

In sum, the present study provides clear electrophysiological evidence showing that a preceding singleton cue with the search-guiding feature can elicit lateralized neural oscillations in both theta and alpha bands. Together with the cue-elicited N2pc component, these lateralized neural activities take effect in different periods of time and might consist of multiple stages of attentional processes during contingent attentional capture. We propose that cue-elicited theta and alpha oscillations are related to global feature enhancement and spatial attention engagement in the process of contingent attentional capture, respectively.

## Data availability statement

The raw data supporting the conclusions of this article will be made available by the authors, without undue reservation.

## Ethics statement

The studies involving human participants were reviewed and approved by the Human Research Ethics Committee, Department of Psychology, Sun Yat-sen University. The patients/participants provided their written informed consent to participate in this study.

## Author contributions

YD and ZQ contributed to the study concept and experiment design. CZ analyzed the data under the instruction and supervision of ZQ and YD. CZ, YD, and ZQ interpreted the data and jointly contributed to the writing of the manuscript. All authors approved the final version of the manuscript for submission.

## Supplementary material

Supplementary Material Supplementary figures for results of induced oscillation amplitude analyses and further analyses.
