# Supplementary material for: Distinct roles of theta and alpha oscillations in the process of contingent attentional capture
Source: Front Hum Neurosci. 2023 Aug 7;17:1220562. doi: 10.3389/fnhum.2023.1220562 (PMC10440541; doi:10.3389/fnhum.2023.1220562)
Supplement: Supplementary Material — Supplementary figures for results of induced oscillation amplitude analyses and further analyses. [file Data_Sheet_1.docx]

**Supplementary Figures**

**
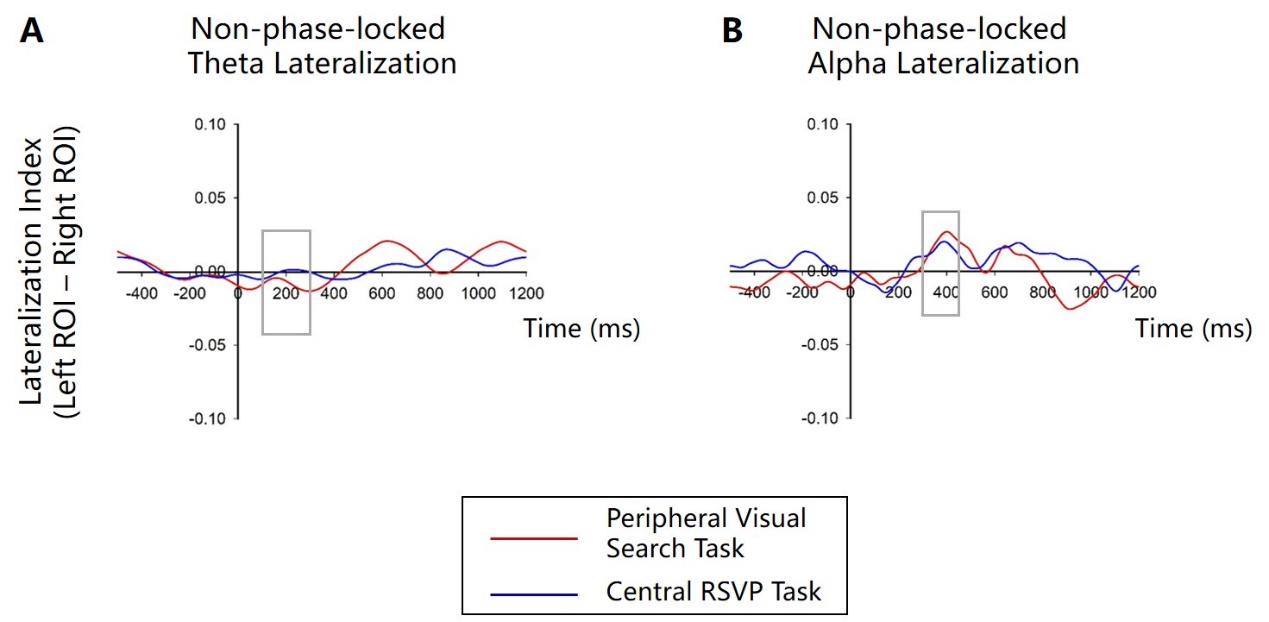
**

**Supplementary Figure 1.** Waveforms of non-phase-locked theta **(A)** and alpha **(B)** lateralization indexes for the peripheral visual search task and the central RSVP task in Experiment 1. Gray boxes show the time windows for analyses.


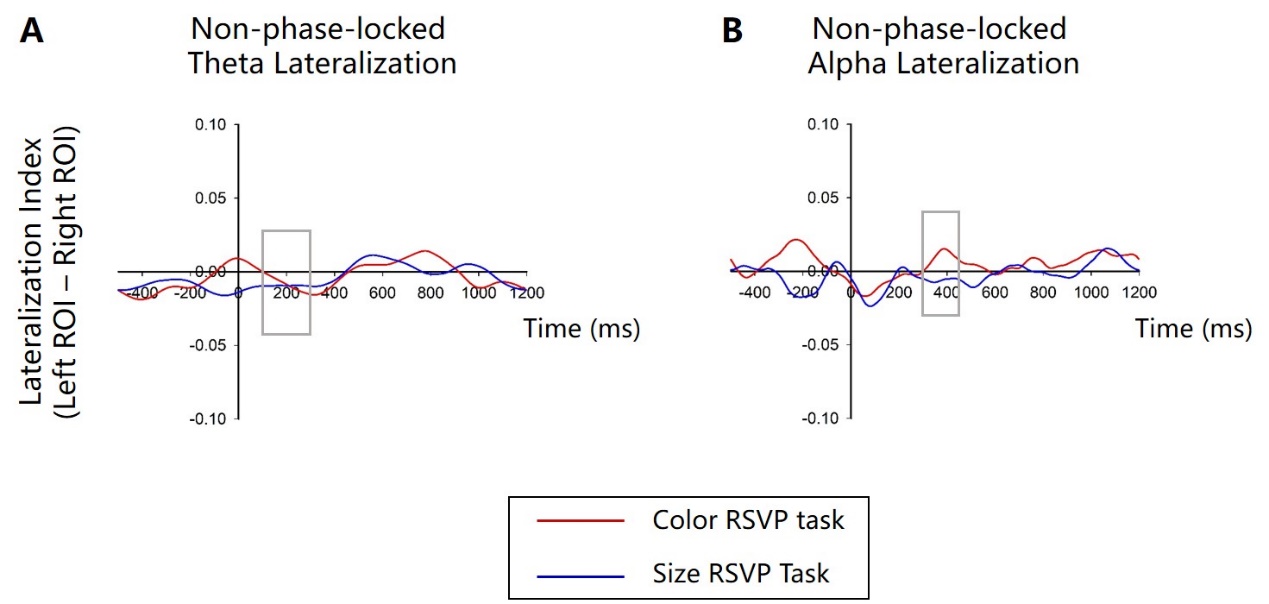


**Supplementary Figure 2.** Waveforms of non-phase-locked theta **(A)** and alpha **(B)** lateralization indexes for the color RSVP task and the size RSVP task in Experiment 2. Gray boxes show the time windows for analyses.


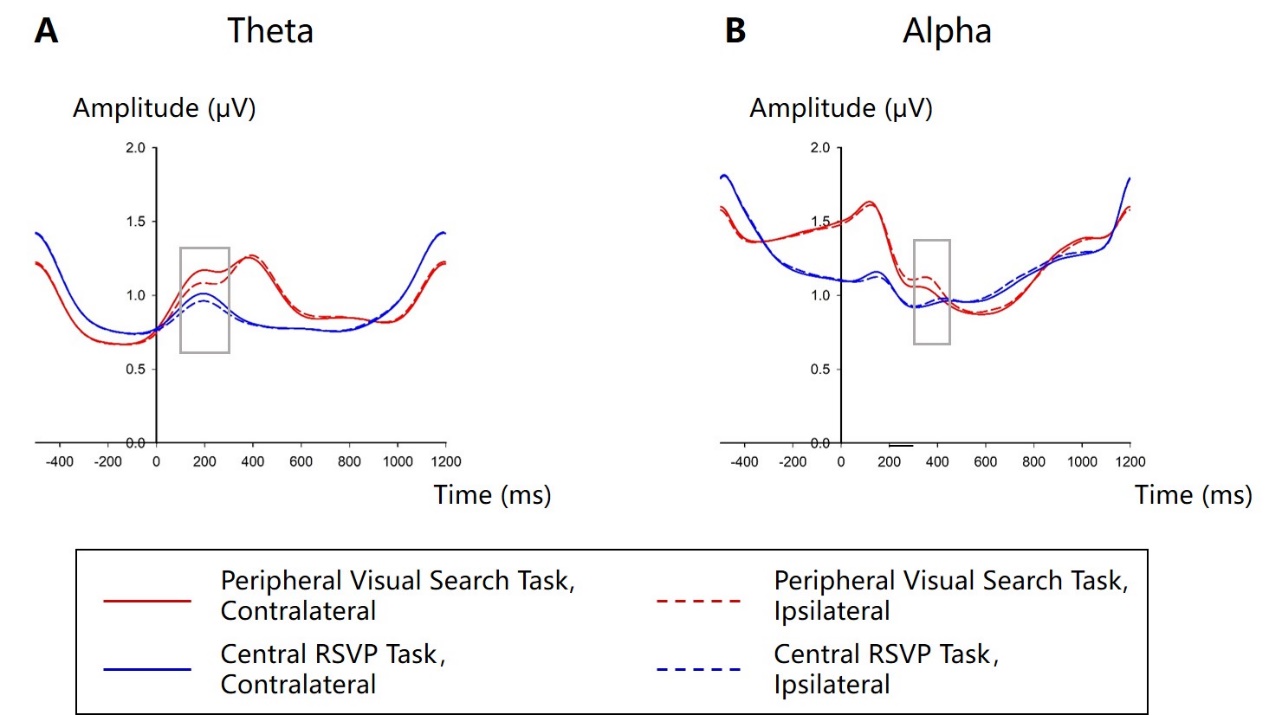


**Supplementary Figure 3.** Waveforms of theta **(A)** and alpha **(B)** amplitudes contralateral and ipsilateral to the singleton cues for the peripheral visual search task and the central RSVP task in Experiment 1. Gray boxes show the time windows for lateralization analyses.


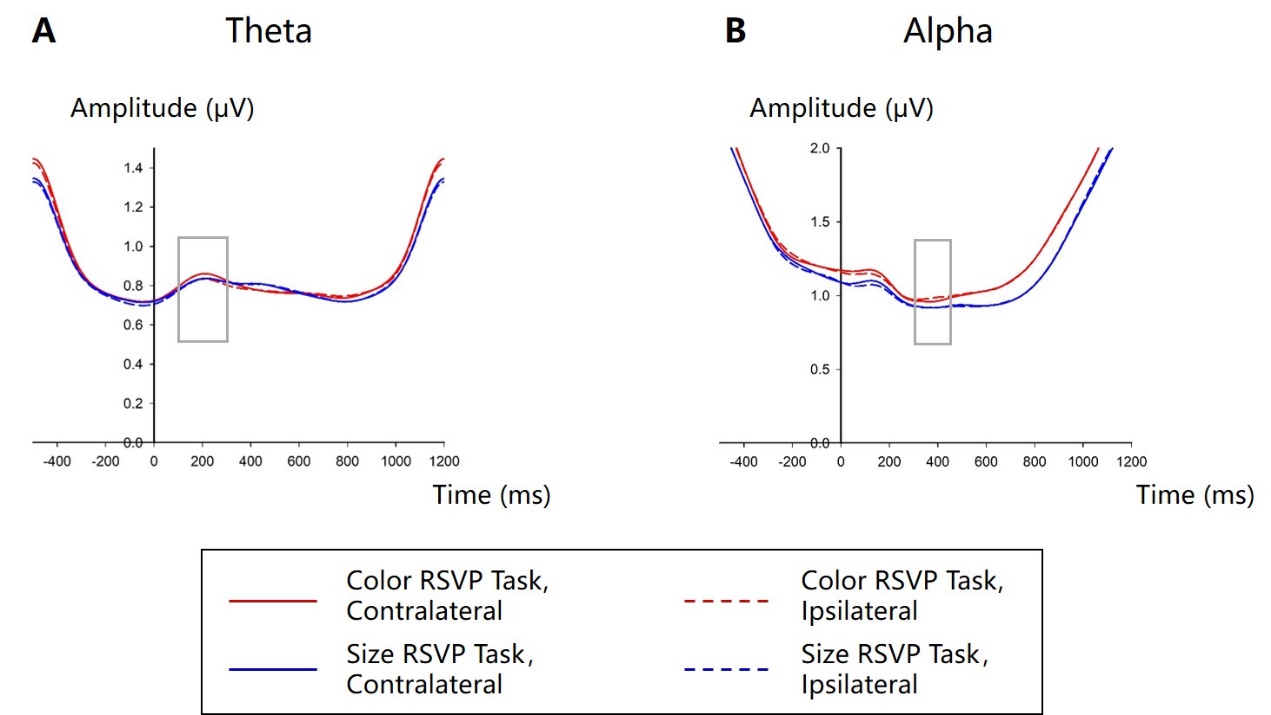


**Supplementary Figure 4.** Waveforms of theta **(A)** and alpha **(B)** amplitudes contralateral and ipsilateral to singleton cues for the color RSVP task and the size RSVP task in Experiment 2. Gray boxes show the time windows for lateralization analyses.


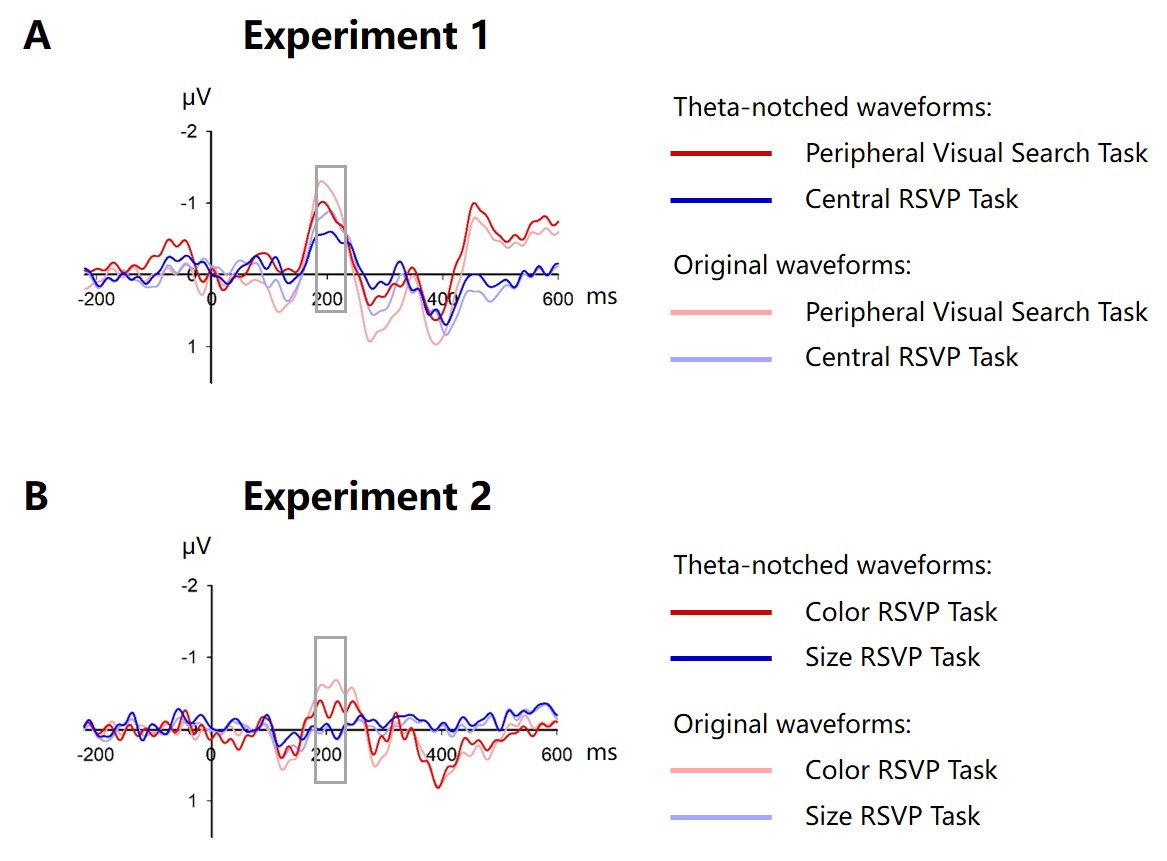


**Supplementary Figure 5.** Contra-minus-ipsilateral ERPs before and after theta band notch-filtering in Experiment 1 **(A)** and Experiment 2 **(B)**. Gray boxes show the time windows for cue-N2pc analyses.


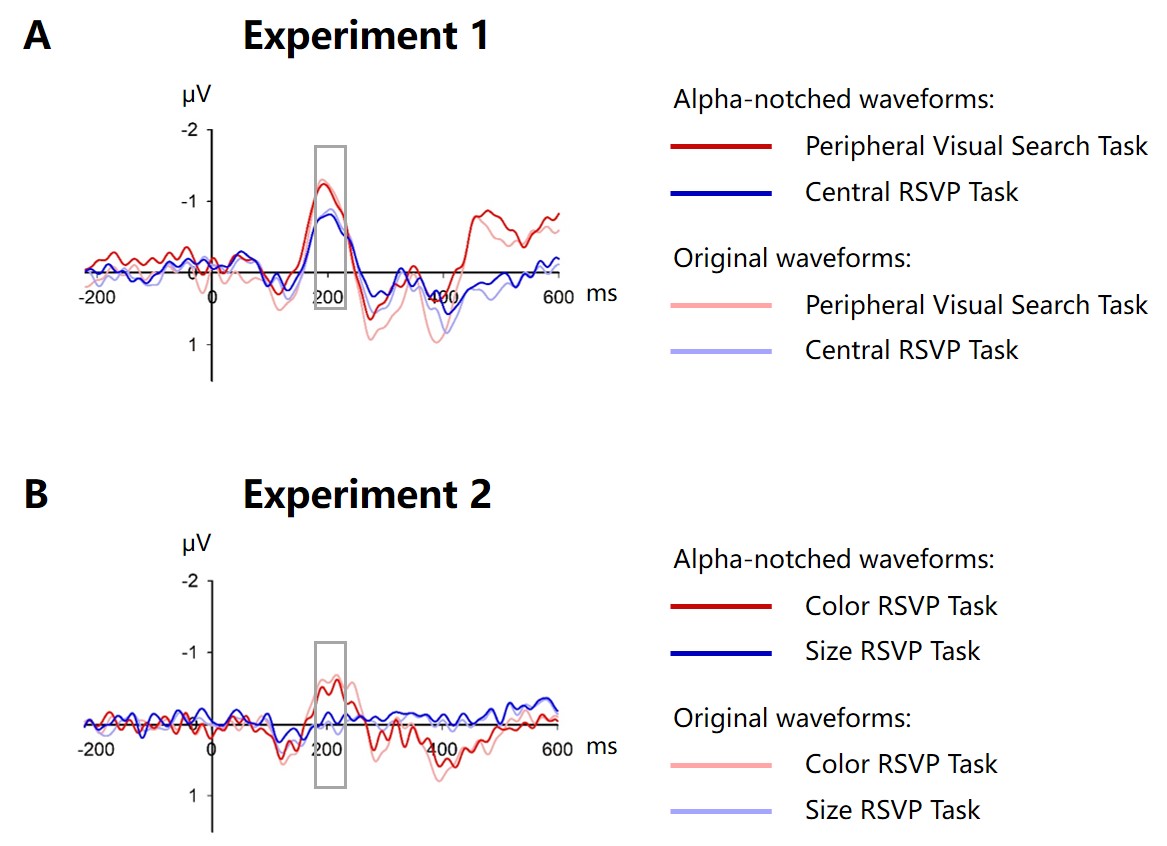


**Supplementary Figure 6.** Contra-minus-ipsilateral ERPs before and after alpha band notch-filtering in Experiment 1 **(A)** and Experiment 2 **(B)**. Gray boxes show the time windows for cue-N2pc analyses.
